# Caregiving Situation as a Predictor of Subjective Caregiver Burden: Informal Caregivers of Older Adults during the COVID-19 Pandemic

**DOI:** 10.3390/ijerph192114496

**Published:** 2022-11-04

**Authors:** Simona Hvalič-Touzery, Marina Trkman, Vesna Dolničar

**Affiliations:** Faculty of Social Sciences, University of Ljubljana, Kardeljeva Ploščad 5, 1000 Ljubljana, Slovenia

**Keywords:** subjective caregiver burden, COVID-19, informal care, quantitative study, Slovenia

## Abstract

The COVID-19 pandemic has created and exacerbated emotional, financial, and technical challenges for informal caregivers of older people. The aim of this study was to explore the caregiving situation and subjective burden of informal caregivers of older family members during COVID-19, and to investigate how a caregiving situation’s characteristics predict the subjective burden of care in times of COVID-19. The study was conducted in April and May 2021 via an online access panel. The sample (n = 612) was determined using a screening test that enabled us to focus on a Slovenian population of informal caregivers aged 40+ caring for a person aged 65+ for at least four hours/week on average. Our findings reveal that the subjective burden of care was high among informal caregivers during COVID-19. Multiple regression analysis showed that the provision of activities of daily living, care duration, average hours of care per week, formal care status, and recipients’ health problems related to dementia or other memory problems significantly predicted the subjective burden of caregivers. These findings call for better recognition of the role of informal caregivers. The time and effort devoted to informal care should be supported by legislation and social security.

## 1. Introduction

In the last two and a half years, the COVID-19 pandemic has reached almost every part of society and affected people in different ways. It has affected people’s physical and mental health [1,2,3,4,5], but it is much more than a health crisis; it is a human, economic, and social crisis that, if not adequately addressed, can lead to an increase in inequality, exclusion, discrimination, and global unemployment [6].

COVID-19 has impacted healthcare systems [7] and the utilization of healthcare services [8,9], demographic trends [9,10], the way people live and socialize, their well-being, and their social development [11]. The consequences of the pandemic are felt in many segments of the population and show adverse effects on people’s lives and well-being [12,13]. The virus has hit the already vulnerable, marginalized, and poorer segments of societies particularly hard, including people living in poverty situations, older adults, persons with disabilities, women, youth, and migrants and refugees [6,14,15]. Older adults are the population with the highest risk of dying from COVID-19, and dependent older adults are also likely to be less capable of supporting themselves in isolation [16]. Studies suggest that during the pandemic, the need for family care has increased, which has affected more women than men [17].

One population group that faced particular difficulties during the COVID-19 outbreak and remained invisible in general discussions of COVID-19 victims was informal caregivers (ICs) [18,19,20,21], who are broadly defined as “persons of all ages who provide care (usually unpaid) to family members, other relatives, partners, friends and neighbors with a long-term illness, disability or other long-lasting health or care need, outside a professional or formal employment framework” [22]. They form the backbone of long-term care systems in many European countries [23,24,25,26], but had not been adequately addressed in many of them even before the pandemic [27,28]. During the pandemic, most countries did not implement specific support for ICs [19,21]. Despite the severe limitations of social and home care services in Slovenia, ICs received very limited support and had to rely largely on their own resources to cope with the new challenges of caring for older family members. The only measure was the one-time solidarity supplement for registered ICs [29]. In addition, a recent study found that almost half of the ICs of older people (48.4%) felt that the Slovenian government had not taken into account the needs of family members helping older people when taking measures to mitigate the effects of the coronavirus, and another 37.4% said that it had barely considered these needs [30].

The EU strategy to support and empower informal carers [22], estimates that over 80% of all long-term care services in Europe are provided by ICs. Furthermore, according to the recent European care strategy for caregivers and care receivers, “the value of hours of long-term care provided by informal caregivers is estimated to be around 2.5% of EU GDP higher than the public expenditure on long-term care” [31]. Yet, policy developments relevant to ICs in EU countries have often been implemented in a fragmented and inconsistent way and have therefore not always led to real improvements in support for ICs. This is particularly true in Slovenia, with its fragmented long-term care system that relies heavily on informal care, and where the needs of ICs are inadequately met [32,33]. Slovenia is a Central Eastern European (CEE) country where care for older people depends mainly on the family, a care model that can be described as “familialistic” [34,35,36]. In Slovenia, familialism is not explicit (i.e., with strong support for family caregivers), but, as in other CEE countries, implicit familialism can be found [34,37]. Family care is assumed but not strongly supported by policy (see, e.g., [38]). Policies on work–life balance do not adequately address the issue of care for older people in terms of inclusive long-term care regulations [29,36]. Thus, we find benefit in a contribution from a post-socialist country with a combination of conservative–corporatist and social-democratic models of care provision [39], along with underdeveloped long-term care systems and strong family elderly care responsibility.

While a comprehensive long-term care system has yet to be implemented, the Slovenian Active Ageing Strategy [40] recognizes the importance of better support for ICs. The recently adopted Slovenian Act on Long-Term Care [41] also recognizes the role of family members; however, it only provides support for a subset of carers, leaving a significant number of ICs unsupported in their informal care. According to the Slovenian Public Opinion Survey 2021 [30,42] conducted during the pandemic, 33.1% of people in Slovenia aged 40 and over provide at least four hours of unpaid informal care per week to a person aged 65 or over, with 43.1% covering the most demanding type of care (basic activities of daily living) [30,42]. Moreover, in 2013, 75.5% of Slovenian people aged 65 or older who received any type of home care received only informal care, 6.7% received only formal care, and 17.8% received a combination of both [33]. A proportion of older people in Slovenia have severe care needs that are unmet [33], placing additional pressure on families to provide care [32].

### 1.1. Aims of the Study

In light of the findings presented in Section 1.2 and 1.3, this study aimed to advance the available knowledge about the caregiving situation and subjective burden of ICs of older people in Slovenia during the COVID-19 outbreak. Although ICs’ subjective burden is well documented in the literature, this is an important topic because it examines how a caregiving situation’s characteristics predict the subjective burden during the pandemic.

Except for one study that included a small sample of Slovenian ICs [20], studies conducted during the pandemic did not include Slovenian ICs, so their situation during the pandemic is largely unknown. Furthermore, until the present study, no comprehensive representative study of the ICs of older adults had been conducted in Slovenia to investigate the caregiving situation and burden of older people’s ICs. While many studies conducted during the pandemic were based on selective and convenience samples (e.g., [43,44]), we conducted a study with a sample from the general population to enable generalization of the results. Therefore, the first aim of the present study, conducted after the first year of COVID-19 in Slovenia, was to determine the caregiving situation and burden during the outbreak. The study targeted a subset of ICs aged 40 to 85 years who provided at least four hours per week of unpaid assistance with activities of daily living (ADL), instrumental activities of daily living (IADL), and/or emotional support to at least one family member aged 65 years or older at the time of the study. ADL is a term that collectively describes activities of functional mobility and personal care, such as bathing, dressing, toileting, eating, dressing, and getting into or out of a bed or chair [45]. IADLs include more complex and demanding activities, such as meal preparation, laundry, managing finances and medication, shopping, doing housework, heavy chores, yard work, or maintenance, transportation, and using the telephone [46]. Similar to Zwar et al. [47], we focused on ICs in this age group because most ICs in Europe and the United States are at least 40 years old [37,42,48,49,50,51,52].

Some gaps in the literature related to the caregiving situation as a predictor of the caregiver’s subjective burden exist. First, many studies have addressed the caregiving situation in relation to a variety of topics (e.g., engagement in advanced care planning, gender studies, and the impact on ICs’ physical and mental health) [47,53,54,55,56]. However, the understanding of caregiving situations varies widely among the studies [57,58,59,60], and they are often vaguely defined [61,62,63,64,65]. Second, the impact of the pandemic on the ICs of older people has been investigated in several studies [43,44,47,66,67,68,69,70], but not the caregiving situation characteristics and subjective burden during the pandemic phase in 2021. Therefore, the second aim of our study was to clearly and holistically define the caregiving situation based on the conservation of resources theory [71], understanding the objective burden [72,73], and the overview presented in Section 1.2, and to investigate how the caregiving situation’s characteristics predict the subjective burden. As mentioned by Flyckt et al. [74], there are different determinants of subjective and objective burdens. Bayen et al. [75] found both similar and distinct predictors for each type. Reinhard and Horrowitz [76], for example, found several predictors of caregiver burden (i.e., factors that may affect the experience of a burden): disruptive behaviors and social support, living arrangements, the relationship between caregiver and care recipient, and demographic characteristics (ethnicity, cultural factors, income, gender, and age). Within these predictors, we focused on those related to the caregiving situation during the COVID-19 pandemic. Our definition of a caregiving situation includes objective burden, defined as the time spent on caregiving and the caregiving tasks undertaken [58,73], as some studies have demonstrated an association between objective and subjective caregiver burdens [77,78,79,80]. The definition also includes some other characteristics of the caregiving situation mentioned in the literature: the health status of the care recipient, the duration of care, formal care resources, and the status of the primary caregiver [57,60,64,65,81].

Two research questions are addressed:

RQ1. What was the caregiving situation and the subjective burden on the ICs of older family members during the COVID-19 outbreak?
RQ2. Which selected caregiving situation characteristics (i.e., weekly hours of care, duration of care, provision of IADL and ADL, health status of the care recipient, type of caregiver, and formal support) significantly predicted ICs’ self-rated subjective burden during the COVID-19 outbreak?

### 1.2. Impact of the Pandemic on ICs

Many international studies have shown that the pandemic has worsened the situation of ICs [12,18,20,25,43,44,67,68,69,82,83,84,85,86,87,88,89,90,91,92]. The pandemic has led to an increase in the intensity of care, the care activities provided, and the burden on ICs [18,20,43,67,70,82,84,85,87,87,88,89,91,92,93,94]. According to a scoping review [91], the pre-existing problems of ICs (e.g., care burden, anxiety, sleep disturbances, coping with a disrupted daily routine, and practical and logistical challenges in accessing health care, social support, and medications) have been exacerbated by the pandemic and restrictive lockdown measures. This has also been confirmed in other recent studies [12,43,86].

In addition, the pandemic has created additional challenges for ICs, such as anxiety and stress related to contracting and spreading the virus, practical concerns related to delayed and discontinued treatment, and a lack of paid formal support services. Most studies report deterioration in the health, quality of life, and psychological well-being of ICs [12,18,20,43,44,83,84,85,87,88,89,90,91,92]. The studies also mention a narrowing of care networks and thus more responsibility for the primary IC [18,92], an increase in the needs of care recipients [20,85], a worsening of the financial situation of ICs during the pandemic [20,83,85,88,89,92,95], an increase in the hours worked by employed ICs or changes in their work patterns [20,85,93,95], and an increase in concern about the situation of care recipients [20,85,89]. According to the Global Carer Well-Being Index [88], based on the 12-country global survey, the pandemic has created three pressure points: emotional, financial, and technological.

Furthermore, since the outbreak of the pandemic, many health and social services provided by formal providers have been closed, reduced, or restructured, leaving older adults at home dependent only on their ICs [18,20,85,89,91]. A study in Europe found that 37.1% of ICs reported experiencing difficulties in accessing and using public or private health and/or social services for their care recipients during the pandemic outbreak, which was rated worst among ICs in Portugal and Germany. More than half of ICs (58.5%) felt that they were not adequately supported by health and social services (with the highest scores in Estonia at 69.9%, Portugal at 65.3%, and Italy at 62.7%) [20].

Several studies have also shown that the impact of the pandemic varied across different subgroups of ICs. In particular, the impact was found to be more severe for female ICs than for their male counterparts in all aspects of the caregiving experience [20,47,83,85,96]. Poorer psychological well-being was associated with ICs who provided intensive care (more than 20 h per week) [70,92], those who provided personal care more frequently than before COVID-19 [14], those who cared for someone with dementia [86,87,97], parental caregivers [18,82], and those who were experiencing financial difficulties [85].

Some studies have specifically addressed the situations of ICs of older people during the pandemic [25,43,44,47,66,67,68,69,70,82]. Of these, however, only a few have addressed the impact of the pandemic on the caregiving burden of ICs of older people [47,70,82,87,94]. For example, a German study [87] that provided insight into the informal caregiving situation during the first wave of the pandemic found that older people’s home care situations deteriorated during the pandemic (e.g., a considerable reduction in the use of support resources such as day care and family physician visits, as well as other support networks such as neighborhood help), which placed a heavy burden on their ICs. They became increasingly concerned about the situation, health, and future of their older relatives and felt more psychologically burdened. A study conducted among ICs during the second wave of the COVID-19 pandemic in Germany found a higher caregiver burden in women than men [47]. A smaller American study [82] of adult children caring for older parents found that the burden of care had increased significantly since the onset of the pandemic, although ICs who had living siblings reported significantly less caregiver burden change than those without siblings. In a study conducted during the third wave of COVID-19 in Japan [70], 41% of ICs complained of an increased caregiver burden. They found that several factors were associated with an increased caregiver burden during the COVID-19 pandemic, which could be due to increased caregiving intensity. However, the researchers also suggested that ICs whose burden increased during the pandemic may have already exhibited risk factors for high caregiver burden prior to its onset. Studies from the Netherlands and Serbia [98,99] found that the declining health of ICs and care recipients during COVID-19 was related to an increased level of perceived burden of care. While several studies have examined specific individual characteristics of caregiving situations during the pandemic [12,18,20,25,43,44,67,68,69,82,83,84,85,86,87,88,89,90,91,92], only one study comprehensively examined the caregiving situation and care involvement [66], and the same study was the only one that examined the caregiving situation in relation to psychosocial burdens.

As this review of existing research shows, the lives of ICs have been affected by a number of factors during the pandemic. However, there is still a gap in understanding how different characteristics of caregiving situation predict the subjective burden of caregiving during the pandemic. In addition, the above studies have not examined Slovenian ICs, so their situation remains largely unknown.

### 1.3. Conceptualization of Caregiver Burden and Caregiving Situation

In the literature, there are various understandings of the concept of caregiver burden, although its multidimensionality is widely recognized [76,100,101,102,103]. Zarit et al. [104] defined caregiver burden as “the extent to which caregivers perceive their emotional or physical health, social life, and financial status as suffering as a result of caring for their relative”. The more recent conceptualization by Liu et al. [105] defines caregiver burden as “the level of multifaceted strain perceived by the caregiver from caring for a family member and/or loved one over time”. The dichotomous conceptualization of burden was developed by Hoenig and Hamilton [106], who separated objective (i.e., events, happenings, and activities) from subjective (i.e., feelings, attitudes, and emotions) measures of burden. This concept has since been widely accepted and applied in a variety of settings. Han and Haley [72] and Montgomery et al. [73] focused on caregiver burden and defined objective burden (OCB) related to caregiving as time spent caring, caregiving tasks assumed, and potential financial problems, whereas subjective burden (SCB) refers to the psychological, social, and emotional effects that caregivers may experience as a result of the objective burden of caregiving. Thus, OCB refers to the observable and tangible burden, whereas SCB refers to personal perceptions and evaluations of the burden [107,108]. We observed that different definitions of SCB and OCB were used in the studies. For example, OCB has been defined using caregiver hours [77], caregiver hours and years [79], expenses and time spent on caregiving [74], and informal care time (hours and days of care) [75]. A variety of measures that vary considerably in length and complexity have been used to measure SCB (e.g., Zarit Burden Interview [109], the CarerQoL VAS [110], the Caregiver Strain Index (CSI) [111], the Caregiver Burden Scale [112], SF-36 Happiness Subscale [113], Modified Caregiver Strain Index (MCSI) [114,115], Caregiver Reaction Assessment (CRA) [116], Appraisal of Caregiving Scale (ACS) [117], Sense of Competence Questionnaire (SCQ) [118], Self-rated Burden (SRB) [108], and Burden Assessment Scale (BAS) [119]). Although the relationship between OCB and SCB has been demonstrated in some studies [77,78,79,80], caution is required because of the different definitions of OCB and SCB. For the purpose of this study, a reliable, accurate, and simple measure of subjective burden—the single-item SRB scale—was used to indicate the degree of subjective caregiver burden.

The caregiving situation has been considered in many studies, but a conceptual definition is lacking, and the understanding of the caregiving situation is less clear. Some studies assess the caregiving situation without providing a rationale for their appraisal of it [61,62,63,64,65,108], and some base their understanding of the caregiving situation on the instrument used to measure it [57,58,60]. We found only one study [81] in which the appraisal of the caregiving situation was based on the conservation of resources theory [71]. In this theory, caregiving conditions refer to objective caregiving situations, such as the older adult’s health (e.g., functional limitations and cognitive impairments) or the time required to provide care. This definition excludes resources (e.g., financial resources, coping, social support, family harmony, and use of services) from the concept of the caregiving situation. Lyons et al. [57] discussed the caregiving situation based on 12 care-related difficulties (e.g., family tension, insufficient financial resources to meet care needs, excessive demands, and seeking community services), in which resources are included in the concept of the caregiving situation. Kraijo et al. [64] examined the caregiving situation from the perspective of objective burden (e.g., duration and intensity of informal care) and the consequences experienced by ICs in providing informal care. Schultz et al. [60] used some items from another study to examine the caregiving situation (e.g., relationship to the care recipient, primary care recipient’s health problem, hours per week providing care, duration of caregiving, and co-residence of caregiver and care recipient).

The understanding of the caregiving situation in the National Alliance for Caregiving & AARP [65] report is very broad and includes demographic information about caregivers and care recipients, the relationship between care recipients and caregivers, the number of people being cared for, and the duration of care. However, the health status of the care recipient, the living situation of the care recipient (e.g., distance from the caregiver), care activities (e.g., provision of ADLs and/or IADLs), intensity of care (e.g., overall duration of care, average hours of care per week), and resources (e.g., formal help, help from family and friends, and status of primary caregiver) are not considered. Common to all of the studies discussed is that their definition of the caregiving situation includes time spent caring (e.g., hours of care per week and/or duration of care) and, in some cases, caregiving tasks undertaken, which overlaps with the definition of objective caregiving burden. However, the caregiving situation goes beyond the definition of the OCB and as can be seen in these examples, sometimes includes the health status of the care recipient. This is discussed by various researchers as important in understanding the caregiving situation [57], as well as formal support for care and possible financial problems. In addition, individual studies add some other variables to the concept of the caregiving situation. Based on this review, we will consider weekly hours of care, duration of care, provision of ADLs and IADLs, health status of the care recipient, status of the primary caregiver, and formal support for care when assessing the caregiving situation.

## 2. Methodology

### 2.1. Study Design, Data Collection, and Sample Description

This study was based on an online survey in which data were obtained through the voluntary participation of respondents. The sample was selected by Valicon from the pool of panel members using quota sampling by statistical region (NUTS-3), gender, and age group. The data were weighted by age, gender, type of settlement, and statistical region according to the population data provided by the Statistical Office of the Republic of Slovenia and the Slovenian Public Opinion Survey.

The data were collected during the COVID-19 pandemic in April and May 2021 via an online access panel. A total of 3284 individuals were invited to participate. Acknowledging the AAPOR standard, we filtered out cases with insufficient data. There were 637 respondents (19.4%) who qualified. The data were then weighted by gender, age, and region to fit the Slovenian population of ICs aged 40 years and over caring for a person who is at least 65 years old for at least four hours/week on average. We obtained 612 eligible respondents. Finally, due to missing values in the variables, we analyzed 549 cases (weighted *n* = 544) using multiple linear regression.

The majority of ICs (68.4%) were between 40 and 64 years old, and 31.6% were 65 years or older. There were slightly more women (51.0%) than men in the sample. A large percentage of ICs were proximate caregivers who lived in the same building or up to half an hour away from the care recipients, while 10.7% of ICs were distant caregivers, living half an hour or more away from their care recipients. The average age of the care recipients was 78.7 years. Of them, 65.5 fell in the last five years (Table 1).

### 2.2. Measures

A structured questionnaire was used. To establish the subjective burden of care, the SRB [108] was used, which expresses burden on a scale ranging from “not at all straining” (0) to “much too straining” (10). We defined ICs’ caregiving situations with care-specific independent variables, such as provision of ADL and IADL, type of carer, overall care duration, average hours of care per week, formal care use, and care recipient’s health problem. Data on caregiving situations were obtained by adapting individual question sets from the Eurofamcare study [120] and the COVID-19 study [20].

### 2.3. Analysis

Descriptive analyses were conducted to examine the characteristics of the participants, their caregiving situations, and their SRB scores. Independent-samples *t*-tests (variables with two categories), and ANOVAs (variables with three or more categories) were performed when analyzing caregiving situation characteristics in relation to SRB total score (RQ1).

Multiple regression analysis was used to examine whether the demographic characteristics of ICs and caregiving situation variables predicted ICs’ SRB scores (RQ2). Forced entry was used as a method of variable entry, so we made no decisions about the order in which a set of independent variables was added to the model. We assessed the model’s fit in several ways. We assessed the R-square and checked whether it indicated a significantly better fit with the model using the predictors than with the model without them by using the F-statistic. We also checked for any multicollinearity among the predictors. All the analyses were conducted using IBM SPSS 22.0.

## 3. Results

### 3.1. Caregiving Situation and SRB during COVID-19

To answer our first research question, we examined informal caregiving situations. Given the different understandings of caregiving situations discussed in Section 1, we describe several indicators of caregiving situations, although not all of them were included in our model. Two-thirds (67.9%) of ICs cared for one older person, 26.7% cared for two persons, and 5.4% cared for more than two older persons. The highest percentage of ICs cared for a parent (53%), 16.6% for their partner, 14.5% for their in-laws, and 15.9% for another family member. A large percentage of ICs (60%) considered themselves to be the primary persons providing assistance to their older relatives. More than half (55.4%) of the ICs provided support with ADLs, while IADLs were provided by the majority of ICs (97.1%) (Table 2). In addition, 70.4% of ICs offered care daily or almost daily, and 27.7% offered care at least once a week. A negligible number offered help less frequently (1.9%). The median amount of informal care provided was 7.1 h per week. A total of 41.4% of ICs offered 11 or more hours of care per week, and 23.3% offered more than 20 h per week. The majority of ICs (73.6%) provided care to an older person for between one and five years, and 15% for up to one year. Several variables measured the health status of the older adults being cared for: 69.1% of them suffered from a chronic disease, 17.4% from dementia, and 7.3% from another mental disorder. A total of 17.8% of care recipients had some form of disability, more than one-third (26.6%) suffered from a temporary disease, and 4.9% had a terminal disease (Table 2). Of the care recipients, 17.6% needed help with most ADLs, one-third (35.1%) needed little help, and slightly less than half (47.3%) got by without help with ADLs. In addition, more than half of the care recipients (52.6%) needed help with most IADLs, 40.7% needed little help, and 6.7% completed IADLs on their own. However, only one-fifth of care recipients (21.1%) received formal help from social or health services in the six months prior (e.g., health care, home help, help with basic care, transportation services, and meal delivery) (Table 2).

SRB scores were measured on a scale ranging from 0 (not at all straining) to 10 (much too straining). The ICs’ average SRB score was 4.78 (SD = 2.92), and 41.2% of ICs scored between 5 and 10. The average SRB score was higher for women than for men (5.06 vs. 4.44), and there were no differences in SRB related to travel time between ICs and care recipients or employment status. Moreover, we examined SRB in the context of informal caregiving situations (Table 2). SRB was higher among ICs who provided ADLs than among those who did not. SRB was higher in primary ICs than in other types of ICs, as well as in ICs whose care recipients had dementia, senility, or other severe memory loss, and in ICs whose care recipients had a terminal illness. The highest burden across all IC subgroups was found in ICs who reported insufficient formal support for their care recipients (Table 2).

Correlational analyses in Table 3 show significant associations between SRB and the following variables: provision of ADLs, average hours of care per week, temporary disease, disability, terminal disease, formal care status indicating that the care recipient receives formal care and does not need any additional help, and formal care status indicating that the care recipient does not receive formal care but would like some.

The results of independent samples *t*-tests and ANOVAs are presented in Table 2.The average SRB scores were significantly different for groups defined by the following variables: formal care status (F = 61.551, *p* < 0.001), provision of ADLs (t = −7.857, *p* < 0.001), duration of care (t = −3.154, *p* < 0.01), and care recipient’s health problems, such as dementia (t = −7.985, *p* < 0.001), terminal disease (t = −3.926, *p* < 0.001) and temporary disease (t = 3.179, *p* < 0.01).

### 3.2. Caregiving Situation’s Variables as Predictors of SRB

To answer our second research question (RQ2), we performed a multiple regression analysis. In Table 4, we present the results of the multiple regression model. Control variables gender of IC (0 = male, 1 = female), travel distance between IC and care recipient (0 = less than 30 min apart, 1 = more than 30 min apart), and employment of IC (0 = unemployed, 1 = employed) are binary variables, while age of IC is a scale variable with values from 40 to 88.

A model containing only the control variables (Model 1 in Table 4) did not significantly improve our ability to predict SRB scores compared to using no predictors, F (4, 539) = 1.372, *p* = 0.242. The control variables of ICs hold weak explanatory power since by themselves they explain just 0.3% of the variance in SRB. Model 2 (Model 1 with additional predictors, namely, provision of ADLs and IADLs, duration of care, and average hours of care per week) explains 15% of the variability in SRB. Model 3 evaluated the effects of all previous variables plus type of carer and formal care status and explained 31.9% of the variability in SRB. Model 4, with all control and caregiving situation variables, explains 33.8% of the variability of SRB. There is a significant improvement in the model’s fit when all variables are included in the model, F (4, 524) = 15.592, *p* < 0.001.

In Model 4, the caregiving situation variables with statistical differences in the means of SRB are: provision of ADL (1 = IC provides at least one ADL, 0 = not providing), care duration (measured in months), average hours of care per week, formal care status (see four categories in Table 2), and recipient’s health problem related to dementia, senility, or other serious memory problem (1 = dementia, 0 = no dementia).

The difference in means between those who provided ADLs and those who did not was B = 0.694 (*p* = 0.002), with those who provided ADLs scoring higher on the SRB scale than those who did not. The variable care duration was recorded as a scale variable with values indicating the number of months from 0 to 576. B = −0.003 (*p* = 0.007) indicates that for every additional month of care, a decrease of 0.003 in the burden score is predicted. Next, the number of average hours of care per week was recorded as a scale variable, with values ranging from 4 to 168. B = 0.020 (*p* < 0.001) indicates that for every additional average hour, the SRB score is significantly higher by 0.020. The caregiving situation variable dementia, senility, or other serious memory problem B = 1.269 (*p* < 0.001) indicates that as we change our perspective from a group of care recipients with dementia to a group of care recipients without it, an increase of 1.269 on the SRB is predicted. Finally, for the caregiving situation variable formal care status, we created categories that combine information about whether the IC’s care receiver receives formal support and whether they require more of it. The reference category for this variable is the third category in Table 2, because it is the most populated category of the four (*n* = 290). We created three dummy variables from the rest of the three categories. The results show that the SRB score was significantly higher in all three comparisons: first, as we change our perspective from the reference category to a category of ICs whose care recipients receive formal care and need additional care (B = 1.333; *p* = 0.001); second, as we change our perspective from the reference category to a category of ICs whose care recipients receive formal care but do not need additional care (B = 3.157; *p* < 0.001); and third, as we change our perspective from the reference category to a category of ICs whose care recipients do not receive formal care but would like some in the future (B = 1.992; *p* < 0.001).

Caregiving situation variables with no statistical differences in the means of SRB scores were provision of IADLs (B = 0.855, *p* = 0.173), whether the care recipient had a temporary disease (B = 0.027, *p* = 0.919), chronic disease (B = 0.172, *p* = 0.499), mental disorder (B = −0.184, *p* = 0.649), terminal disease (B = 0.808, *p* = 0.115), or disability (B = −0.092, *p* = 0.756), and type of carer. For the categorical variable type of carer (1 = primary, 2 = secondary, 3 = shared), we chose the reference category as number 1 (*n* = 326) and created two dummy variables for categories 2 and 3. Our results show that secondary ICs (B = −0.519, *p* = 0.127) and shared care (B = −0.387, *p* = 0.115) had non-significant differences in average SRB scores when compared to primary ICs. In line with expectations, all four control variables—gender of IC (B = 0.243, *p* = 0.246), travel distance between IC and care recipient (B = 0.109, *p* = 0.748), employment status of IC (B = 0.475, *p* = 0.086), and age of IC (B = 0.004, *p* = 0.769)—had differences in group means that were not statistically significant. We checked for multicollinearity issues by examining the values of the variance inflation factor (VIF). VIF indicates whether a predictor has a strong linear relationship with the other predictors. The values were between 1.051 and 1.826, which is acceptable [122].

## 4. Discussion

The current analysis provides new insights into the characteristics of the caregiving situation of ICs of older people during the COVID-19 pandemic. Characteristics of the caregiving situation of ICs were studied also as predictors of subjective care burden, as measured on the SRB scale. While there is a lack of data linking specific contexts, care recipients’ characteristics, and the burden of ICs [123], this study attempts to fill this research gap. Two important findings emerged from this analysis regarding ICs’ SRB and can be related to the research questions: (RQ1) better understanding of caregiving situation and SRB during the COVID-19 in Slovenia; and (RQ2) the variables of the caregiving situation in our model (provision of ADLs, duration of care, average hours of care per week, caring for persons with dementia, and formal care status) explained 36.1% of the variability in SRB scores.

Our study showed a complex picture of caregiving situation in Slovenia during the pandemic, with more than half of ICs providing ADLs, a third of ICs providing care to more than one older person, a high percentage (70%) of ICs providing care daily or almost daily, and three quarters of ICs providing care for more than one year. As shown in another Slovenian study [34] conducted a few years before the COVID-19 pandemic, formal care was used by a small proportion of informal care recipients, resulting in a substantial care burden for ICs years before the pandemic. As shown in a scoping study by Muldrew et al. [91], the pandemic exacerbated existing issues related to informal caregiving and caused additional issues. In our study, the ICs of older people who provided more demanding and intensive care experienced a higher subjective burden of care. These findings are consistent with the results of several other studies [18,43,66,70,91,99,124,125] that found a high and increased caregiver burden during the COVID-19 pandemic. A German study of 1000 ICs [66] found that 25.5% to 39.7% of ICs reported that their caregiving situations somewhat or greatly worsened during the COVID-19 pandemic, especially among those caring for a person with dementia or who normally relied on professional help. Our study also confirmed a higher subjective burden among the ICs of people with dementia.

Multiple regression analysis showed that two variables, the formal care status of the care recipient, and caring for a person with dementia or other severe memory loss, are the two strongest predictors of SRB. Provision of ADLs, longer duration of care, and higher average hours of care per week also predicted SRB, but to a lesser extent. These results are consistent with the findings of a Japanese study on ICs [70]. The authors of the Japanese study found that factors associated with increased caregiver burden during the COVID-19 pandemic were ICs with depressive symptoms, care receivers with dementia, care receivers with low scores for ADL performance, care days and times, and the use of home care and visiting care services. Furthermore, a German study by Bergmann and Wagner [18] found that ICs who cared for their parents during the first phase of the pandemic in spring/summer 2020, and who increased the frequency of personal care, reported significantly more psychological distress (e.g., sadness, depression, anxiety, and nervousness). According to Rajovic et al. [99], caregiver burden is a mediating factor between determinants of the caregiving process (ADL, duration of care, level of care complexity, social support, financial support, and physical health) and indirect effects on depressive symptomatology. A cross-sectional study on ICs in 41 Serbian municipalities during the first phase of the pandemic found that more than two-thirds (71.9%) of ICs in Serbia experienced a mild to severe burden, and more than one-quarter (27.1%) had mild to severe depression symptomatology. They found that functioning capacity in ADL was one of the most important predictors of IC burden. Other predictors of IC burden were the duration of daily care, the level of care complexity, insufficient financial support, and ICs’ self-perceived physical health. A study of 2468 ICs in several European countries [20] and a Lithuanian study of ICs [124] found that women caregivers were significantly more burdened than men. This was in contrast to our study and a German study of ICs of older adults in the second wave of COVID-19 [47], which found no difference in caregiver burden between genders. However, they did find higher levels of anxiety and lower quality of life among female ICs than among male ICs. Cohen et al. [125] found that increases in the burden of care in the early months of the pandemic were associated with increased intensity of care. They also found that changes in care intensity and care burden were complex and varied by gender. Interestingly, they found that men with higher initial care intensity were more likely to have an increase in care burden due to the pandemic, but the association was not significant for women.

According to the OECD working paper [19], ICs “are the first line of support for older people in many countries and COVID-19 and responses have exacerbated challenges for caregivers, as many of them had to provide care in a context of limited availability of long-term care services”, which was also noted in our study. Therefore, they should be identified as a priority group within the long-term care sector and receive direct support for their informal care [19,91]. In addition, they should be legally recognized and, as such, have access to a range of rights (e.g., social protection, financial support, pension, respite care, information, training for better care, flexible work arrangements, and care allowances) [19,22,91]. Public authorities need to promote a policy framework for the creation of ICT-based solutions for ICs and beyond, as European policy has recognized the role of these technologies in supporting aging in place [126,127,128] and ICs [129,130]. Several studies [130,131,132,133,134] have identified a high potential for ICT-enabled solutions to improve the well-being of ICs and reduce their care burdens, and these should be made available to ICs and older people.

As mentioned, Slovenia has a familialistic model of care, where the family is not only normatively but also legally obliged to help its older family members. At the same time, ICs receive minimal support. The weakness of this system became even more apparent during the COVID-19 pandemic, when limited access to home care and health services forced families to take on even more responsibility for care. As expected, the results showed a high level of caregiving intensity and self-rated burden among family members during the pandemic. The results also showed that the formal care status of the care recipient (whether or not they receive sufficient formal support), caring for a person with dementia or other severe memory loss, and high care intensity (longer duration of care and higher average hours of care per week) were relatively strong predictors of self-rated burden. As mentioned, a high care burden and lack of support for ICs can have very negative consequences for ICs and, in the long run, for the long-term care system. These results call for urgent changes in Slovenian long-term care policy and demonstrate the need to support ICs. We can say that the important lesson from the pandemic, also highlighted in this study, is the need to better recognize and value informal care in Slovenia. The results of this study, as well as a recently presented European care strategy that provides a framework for improving the situation for both those in need of care and the people who care for them professionally or informally and that also emphasizes the importance of supporting ICs [31], will hopefully bring some improvements for ICs in Slovenia.

### Limitations and Future Research Directions

Our results should be considered in the context of the study’s limitations. First, caution should be exercised in interpreting the results, as they are applicable only to this sample of ICs in Slovenia. Slovenian ICs may have a caregiving situation that is so specific to the country of data collection that the results cannot be easily generalized to ICs in other countries (e.g., a strong reliance on family for social support and a large proportion of ICs living in multigenerational households) [35]. Second, this was a cross-sectional study, and it is not known whether the caregiving situation and SCB are similar over time, especially before and after the pandemic. It is also not possible to determine temporal relationships to estimate SCB. Because most research in the area of informal care outcomes is based on cross-sectional analyses [135], future longitudinal studies should examine caregiving outcomes over time.

Third, our model was tested on a general population of ICs aged 40 to 85 years who provided at least four hours per week of unpaid assistance with ADLs, IADLs, and/or emotional support to at least one family member aged 65 years or older. Although gender, employment, age, and travel distance between IC and the care recipient were included as control variables in the regression model, the analysis could be improved if the model were tested on different subgroups of the heterogeneous group of ICs. Indeed, there could be significant differences in caregiving situations and SCB between different subgroups of ICs defined by different sociodemographic characteristics. In particular, the oldest age groups of ICs should be studied, as ICs aged 75+ are likely to become one of the fastest growing segments of the caregiving population in coming years [25]. Future studies could therefore “oversample older age groups, in order to have crucial information about this—for long-term care services very important—target group” [25]. Further qualitative studies could also examine specific subgroups of ICs, so that profound nuances of the caregiving situation and SCB can be explored.

Fourth, only a limited number of predictors of SCB were examined in this study. Although we included important characteristics of the caregiving situation, we could also add, for example, characteristics of the relationship between IC and the care receiver, support received for caring activities from the ICs’ social network, number of people cared for, ICs’ financial resources, caregiver–care recipient co-residence, and frequency of caregiving activities. In this way, the model could explain an even higher percentage of the variance in the dependent variables. However, in deciding which predictors to include in the exploratory regression model, a balance must be struck between being too narrow and too broad. We have attempted to include a wide range of predictors identified in various studies to gain a basic understanding of how they could significantly predict the SCB of ICs.

Finally, future analysis could be enriched by performing a path analysis with additional dependent variables, such as physical health (e.g., spinal disorders and pain conditions) and mental health (e.g., depressive symptoms, anxiety, and stress), difficulties in social life, labor market participation and social inclusion, and quality of life in general. In addition, the SCB of ICs could be conceptualized and measured differently, taking into account physical, social, and financial burdens.

Considering the above limitations and possible future research directions, it would be worthwhile to conduct a longitudinal study and/or a cross-national comparative study in the future. A comparison group with non-caregivers from population-based samples could also be introduced in future studies to further investigate predictors of health-related quality of life.

## 5. Conclusions

Many studies related to the outcomes of informal caregiving included care receivers with a specific condition (e.g., dementia, cancer, or mental or physical disorders) and from caregiving arrangements or situations. Many have also recruited only specific subgroups of caregivers (e.g., female or spousal). Most studies do not assess an individual appraisal of the ICs regarding their caregiving situations, or their motivations and outcomes [135]. As a response to this, our study is based on data that fit the Slovenian population of ICs aged 40 years and over. It is also focused on care receivers aged 65 and older with different conditions, characteristics, and situations. While many studies are based on selective and convenience samples, we conducted a study with a sample from the general population to be able to generalize the results. This is also one of the few national studies focusing on caregiving situation characteristics and SCB during the pandemic phase in 2021. An original feature of this study is that it is the only empirical study examining the caregiving situation characteristics as predictors of SCB during the pandemic. Empirical analysis showed a high caregiver burden during the COVID-19 pandemic. The caregiving situation variables that significantly predicted SRB are: provision of ADL, care duration (measured in months), average hours of care per week, formal care status, and recipients’ health problems related to dementia, senility, or other serious memory problems. Further conceptual and empirical research is needed to uncover the underlying mechanisms of the interplay between the characteristics of the caregiving situation (which includes OCB) and SCB, as well as the relationship between OCB and SCB.

## Figures and Tables

**Table 1:** Characteristics of informal caregivers and their care recipients.

Categories	Total
Informal caregivers’ characteristics (*n* = 544)	
Gender (% female)	51.0
Age (mean [standard deviation])	57.63 [10.85]
Education ^1,2^	
Primary school (ISCED Levels 0–2, %)	2.6
Secondary school (ISCED Level 3, %)	51.4
Higher education (ISCED Levels 5–8, %)	46.0
Employment status	
Employed (%)	54.3
Retired (%)	36.9
Unemployed (%)	8.8
Married or has a partner (% yes)	80.0
Time distance between IC and care recipient	
Live in in the same building (%)	54.8
Live up to 30 Min away (%)	34.4
Live 30 Min or more away (%)	10.8
Care recipients’ characteristics	
Age (mean [standard deviation])	78.68 [8.50]
Poor or very poor health (% yes) ^3^	27.7
Fall experience in the last 5 years (% yes)	65.5

Note: ^1^ We mapped Slovenia’s educational levels with ISCED’s levels with help from the following governmental document [121]; ^2^ The share of missing values per variable was 0.4%; ^3^ The share of missing values per variable was 0.1%.

**Table 2:** Caregiving situation and self-rated care burden.

Caregiving Situation’s Variables	N = 544 (Weighted)	SRB (Mean (Standard Deviation))	*t*-Test or F-Statistics ^4^
Provision of ADLs ^1^			
1 = Providing (%)	55.4	5.59 (2.98)	−7.757 ***
0 = Not providing (%)	44.6	3.77 (2.51)	
Provision of IADLs ^2^			
1 = Providing (%)	97.1	4.79 (2.92)	−0.676 ^ns^
0 = Not providing (%)	2.9	4.28 (3.11)	
Type of carer			
1 = Primary (%)	60.0	4.94 (2.92)	1.599 ^ns^
2 = Secondary (%)	12.8	4.66 (3.02)	
3 = Carers sharing care responsibility equally (%)	27.2	4.44 (2.85)	
Duration of care (months; mean (standard deviation))	69.68 (92.62)		
Average hours of care per week (mean (standard deviation))	18.29 (23.24)		
Formal care status			
1 = Uses formal support ^3^ and does not need more of it (%)	8.3	5.21 (3.14)	61.551 ***
2 = Uses formal support and would like more of it (%)	12.9	7.27 (2.43)	
3 = Does not use formal support nor wants any (%)	53.3	3.47 (2.37)	
4 = Does not use formal support but would like it (%)	25.5	6.09 (2.68)	
Care recipient’s health problem			
1 = Has temporary disease (%)	26.6	4.12 (2.80)	3.179 **
0 = Does not have temporary disease (%)	73.4	5.01 (2.93)	
1 = Has chronic disease (%)	69.1	4.73 (2.85)	0.564 ^ns^
0 = Does not have chronic disease (%)	30.9	4.88 (3.08)	
1 = Has dementia ^4^ (%)	17.4	6.71 (2.53)	−7.985 ***
0 = Does not have dementia ^4^ (%)	82.6	4.37 (2.84)	
1 = Has mental disorder (%)	7.3	5.40 (3.13)	−1.405 ^ns^
0 = Does not have mental disorder (%)	92.7	4.73 (2.90)	
1 = Has disability (%)	17.8	5.63 (3.19)	−3.212 ^ns^
0 = Does not have disability (%)	82.2	4.59 (2.83)	
1 = Has terminal disease (%)	4.9	6.91 (2.59)	−3.926 ***
0 = Does not have terminal disease (%)	95.1	4.66 (2.90)	

Note: ^1^ Activities of daily living; ^2^ Instrumental activities of daily living; ^3^ Care recipient has received formal services, defined as social and home health care provided by qualified personnel (e.g., health professionals, social workers, and social care attendants) in the past six months; These services include nurse visits, social home help, home-delivered meals, and personal care assistance; ^4^ This refers to people with dementia, senility, or other serious memory problems; For variables with two categories, we ran independent samples *t*-tests, while for variables with three or more categories, we ran an analysis of variance and reported F-statistics; *** *p* < 0.000; ** *p* < 0.01; *p* < 0.05; ns = non-significant.

**Table 3:** Pearson correlations among caregiving situation characteristics’ variables.

Caregiving Situation’s Variable	1	2	3	4	5	6	7	8	9	10	11	12	13	14	15	16
1. ADLs	1															
2. IADLs	−0.022	1														
3. Care duration	−0.097 *	−0.024	1													
4. Average hours of care per week	0.179 *	0.040	0.059	1												
5. Formal care status: reference category vs. care recipient receives formal care and would like to get additional care ^2^	0.087 *	−0.043	−0.041	−0.012	1											
6. Formal care status: reference category vs. care recipient receives formal care and does not need any additional help ^2^	0.215 *	−0.082 *	0.029	0.068	−0.115 *	1										
7. Formal care status: reference category ^1^ vs. care recipient does not receive formal care but would like to get some ^2^	0.120 *	0.025	−0.049	0.084 *	−0.175 *	−0.226 *	1									
8. Type of carer: primary vs. secondary ^1^	−0.049	−0.044	0.000	−0.148 *	0.097 *	0.064	0.000	1								
9. Type of carer: primary vs. sharing equal responsibility ^1^	−0.085 *	−0.031	−0.089 *	−0.105 *	0.018	−0.053	0.043	−0.234 *	1							
10. Care recipient has temporary disease	−0.091 *	0.010	−0.076 *	−0.071 *	0.005	−0.179 *	−0.107 *	−0.071 *	0.045	1						
11. Care recipient has chronic disease	−0.073 *	0.028	0.028	−0.010	−0.053	−0.034	0.074 *	−0.069	−0.003	−0.330 *	1					
12. Care recipient has dementia	0.063 *	0.021	−0.106 *	0.75 *	0.033	0.113 *	0.192 *	0.040	−0.003	−0.148 *	−0.217 *	1				
13. Care recipient has mental disorder	0.043	0.048	0.010	−0.004	−0.008	0.074 *	0.093 *	−0.083 *	−0.013	0.062	−0.011	0.081 *	1			
14. Care recipient has disability	0.262 *	0.063	0.105 *	0.187 *	0.033	0.221 *	0.057	−0.034	−0.014	−0.0138 *	−0.053	0.062	0.011	1		
15. Care recipient has terminal disease	0.145 *	−0.100 *	−0.080 *	0.125 *	0.063	0.138 *	0.051	0.094 *	−0.020	−0.112 *	−0.142 *	0.000	−0.064	−0.058	1	
16. Self-reported burden of care (SRB)	0.311 *	0.029	−0.134	0.244 *	0.045	0.329 *	0.265 *	−0.015	−0.070	−0.135 *	−0.024	0.303 *	0.060	0.137 *	0.166 *	1

^1^ Primary = 0; ^2^ Reference category = care recipient does not use formal support nor wants any = 0; * *p* < 0.05.

**Table 4:** Multiple regression analyses predicting ICs’ subjective burden during COVID-19.

		Model 1 (Partial Model)		Model 2 (Partial Model)		Model 3 (Partial Model)		Model 4 (Full Model)	
		B	Beta	B	Beta	B	Beta	B	Beta
Control variables	Gender	0.579 *	0.099	0.486 *	0.083	0.266	0.046	0.243	0.042
	Travel distance between IC and care recipient	0.026	0.003	0.265	0.028	0.060	0.006	0.109	0.012
	Employment	0.125	0.021	0.574	0.098	0.532	0.091	0.475	0.081
	Age	0.004	0.014	0.006	0.022	0.005	0.018	0.004	0.014
Informal caregiving situation’s variables	Provision of ADLs			1.568 ***	0.267	0.777 **	0.132	0.694 **	0.118
	Provision of IADLs			0.449	0.026	0.816	0.047	0.855	0.049
	Care duration			−0.004 **	−0.116	−0.004 **	−0.119	−0.003 **	−0.098
	Average hours of care per week			0.028 ***	0.220	0.022 ***	0.177	0.020 ***	0.163
	Type of carer: primary vs. secondary					−0.422	−0.048	−0.519	−0.059
	Type of carer: primary vs. sharing equal responsibility					−0.374	−0.057	−0.387	−0.059
	Formal care status: reference category ^1^ vs. care recipient receives formal care and would like more					1.484 ***	0.140	1.333**	0.126
	Formal care status: reference category ^1^ vs. care recipient receives formal care and does not need additional help					3.400 ***	0.391	3.157***	0.363
	Formal care status: reference category^1^ vs. care recipient does not receive formal care but would like some					2.253 ***	0.337	1.992***	0.298
	Care recipient has temporary disease							0.027	0.004
	Care recipient has chronic disease							0.172	0.027
	Care recipient has dementia							1.269 ***	0.165
	Care recipient has mental disorder							−0.184	−0.016
	Care recipient has disability							−0.092	−0.012
	Care recipient has terminal disease							0.808	0.060
	F-statistic	1.372		12.966		20.610		15.592	
	Sig. of F-statistic	0.242		0.000		0.000		0.000	
	R-square	0.010		0.162		0.336		0.361	
	Adjusted R-square	0.003		0.150		0.319		0.338	

Note: N = 544. B = unstandardized coefficients; beta = standardized coefficients; ^1^ ICs whose care recipients do not receive formal care and do not need any; * *p* < 0.05; ** *p* < 0.01; *** *p* < 0.001; Source: author’s analysis based on original data.

## Data Availability

The dataset is available in the PsychArchives repository (https://www.psycharchives.org/); http://dx.doi.org/10.23668/psycharchives.8240 accessed on 1 November 2022.
